# Current rectifying and resistive switching in high density BiFeO_3_ nanocapacitor arrays on Nb-SrTiO_3_ substrates

**DOI:** 10.1038/srep09680

**Published:** 2015-04-08

**Authors:** Lina Zhao, Zengxing Lu, Fengyuan Zhang, Guo Tian, Xiao Song, Zhongwen Li, Kangrong Huang, Zhang Zhang, Minghui Qin, SujuanWu, Xubing Lu, Min Zeng, Xingsen Gao, Jiyan Dai, Jun-Ming Liu

**Affiliations:** 1 Institute for Advanced Materials and Guangdong Provincial Key Laboratory of Quantum Engineering and Quantum Materials, South China Normal University, Guangzhou 510006, China; 2 Department of Applied Physics, The Hong Kong Polytechnic University, Hong Kong, China; 3 Laboratory of Solid State Microstructures and Innovation Center of Advanced Microstructures, Nanjing University, 210093, China

## Abstract

Ultrahigh density well-registered oxide nanocapacitors are very essential for large scale integrated microelectronic devices. We report the fabrication of well-ordered multiferroic BiFeO_3_ nanocapacitor arrays by a combination of pulsed laser deposition (PLD) method and anodic aluminum oxide (AAO) template method. The capacitor cells consist of BiFeO_3_/SrRuO_3_ (BFO/SRO) heterostructural nanodots on conductive Nb-doped SrTiO_3_ (Nb-STO) substrates with a lateral size of ~60 nm. These capacitors also show reversible polarization domain structures, and well-established piezoresponse hysteresis loops. Moreover, apparent current-rectification and resistive switching behaviors were identified in these nanocapacitor cells using conductive-AFM technique, which are attributed to the polarization modulated *p-n* junctions. These make it possible to utilize these nanocapacitors in high-density (>100 Gbit/inch^2^) nonvolatile memories and other oxide nanoelectronic devices.

BiFeO_3_ (BFO) has attracted intensive attention in the past decades due to its well-known room temperature multiferroicity, in addition to excellent ferroelectric, magnetoelectric, photovoltaic, and electromechanical properties, offering a series of promising applications in high density memory, photovoltaic, electromechanical, and spintronic devices^1^,^2^,^3^,^4^,^5^. With the current trends of high integration and miniature in semiconductor industry, nanoscale multiferroic/magnetoelectric materials (e.g. BFO) as cutting-edge nano-electronic research field have been receiving more and more attention.

It was reported that BFO nanodots exhibit various unique properties inaccessible otherwise^6^,^7^,^8^,^9^,^10^. For instance, BFO nanoparticles show strong size-dependent magnetic and photocatalytic properties due to the size confinement^7^,^8^. In free-standing tetragonal-like BFO nano-islands, an unexpected shape memory behavior in association with a martensitic-like phase transformation was observed, owing to the release of substrate clamping^11^. Isolated nanocapacitor structures can also greatly enhance the stability of the ferroelastic domain switching in BFO, enabling the electric control of antiferromagnetism^12^. However, for large scale integration devices, high density capacitor array is very essential, nevertheless up to now there have been yet very few reports addressing both their fabrication process and nanoelectronic properties^13^. On the other hand, the electrical properties of nanocapacitor also critically depend on their electrodes, which not only affect the uniformity of electric field distribution inside the capacitors, but also change their piezoelectric and transportation behaviors via interfacial barrier modulation^14^,^15^. For instance, we have demonstrated in a previous work that a unique complex domain structure along with an enhanced resistive switching behavior, can be introduced by self-assembled Bi_2_O_3_ nanoisland top electrodes in ultrathin BFO film-based nanocapacitors^15^.

In this work, we have developed well-ordered BFO nanocapacitor arrays with SrRuO_3_ (SRO) top electrodes on conductive Nb-SrTiO_3_ (Nb-STO) substrates by the anodic alumina (AAO) template assisted method. We have selected Nb-STO as bottom electrode as it has been reported to significantly improve the resistive switching behavior of ultrathin ferroelectric BaTiO_3_ film^16^. The nanocapacitor arrays have well-epitaxial structure, showing apparent ferroelectric polarization and interesting current rectifying resistive switching characteristics. They are promising for applications in ultrahigh density recording devices.

## Results and discussion

**Figure 1:**
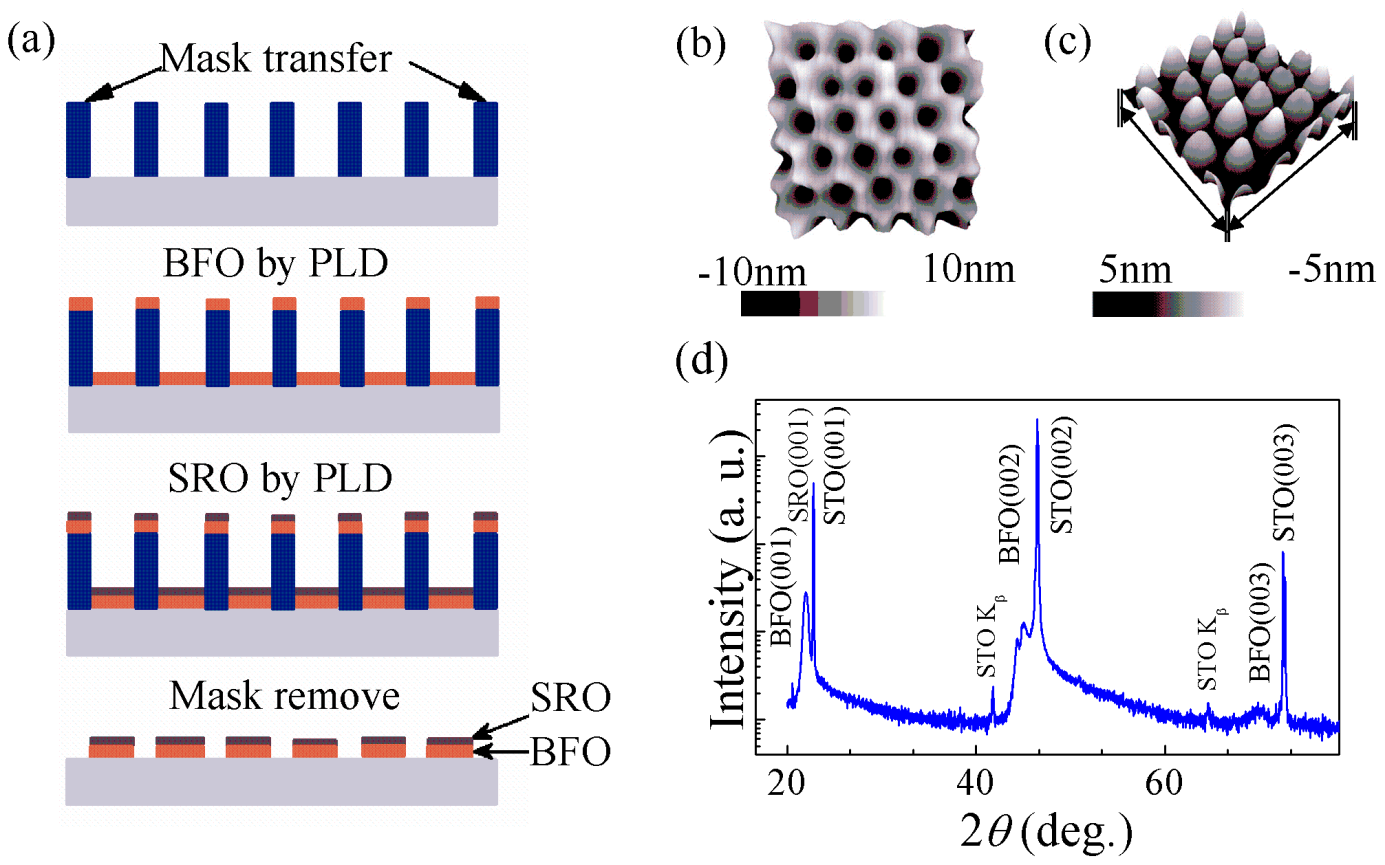
Fabrication details for the nanocapacitor arrays. (a) Schematic flow chart illustrating the fabrication procedure for the BFO-SRO nanocapcitor arrays on Nb-STO substrate; (b, c) three-dimensional topographic image for the AAO mask (b) and BFO/SRO nanodots (c); (d) XRD diffraction pattern for the as-deposited SRO/BFO/Nb-STO nanodot heterostructures.

To construct the nanocapacitor array, we have deposited BFO/SRO heterostructured nanodots on conductive Nb-STO substrates which serve as bottom electrodes. In brief, the BFO/SRO herterostructured nanodots were sequentially grown on a conductive Nb-doped (100) SrTiO_3_ (Nb-STO) substrate through an AAO template by pulsed laser deposition (PLD). The fabrication process is illustrated in Fig. 1(a), the details of which will be presented in the method section, and further information can also be found in our previous reports^17^,^18^. As shown Fig. 1(c), the BFO nanodots exhibit an average lateral size of ~60 nm, and a dot-dot distance of ~120 nm. The XRD spectrum shows a (001)-orientated BFO/SRO heterostructure on Nb-STO, as reflected by the (00*l*) diffraction peaks shown in Fig. 1(d).

**Figure 2:**
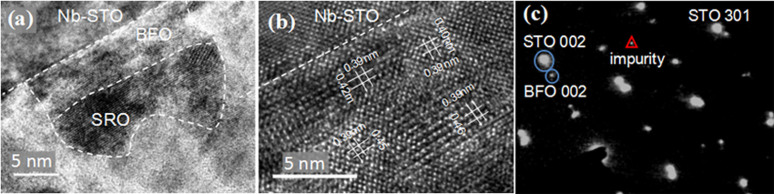
Cross-section TEM images for the SRO/BFO/Nb-STO nanodot heterostructure. (a) Relative smaller magnification image as a overview for a nanocapacitor structure, and (b) larger magnification image; (c) selected area electron diffraction (SAED) alone the <010> direction, showing diffraction spots of STO, BFO, and SRO, along with minor impurity phases.

The Nb-STO/BFO/SRO nanoscale features were also examined by the cross-section TEM observations shown in Fig. 2. The cross-section image demonstrates that each cell is composed of well-epitaxial BFO thin layer of ~10 nm in thickness, covered with a SRO capping layer as top electrode. It is also worthy of mention that the interface between BFO and SRO nanodots is not very flat, which is limited by our fabrication method. The individual layers were also checked by Energy Dispersive X-Ray Spectroscopic Analysis (EDX). From the transmission electron microscopy (TEM) cross-section images, it is seen that both BFO and SRO show well-established single crystalline with the same orientation as the substrate. The epitaxial quality was further examined by selected area electron diffraction (SAED) along the <010> direction (Fig. 2(c)), where apparent diffraction spots of STO/SRO and BFO, along with minor amount of impurity phase, can be identified. It should be mentioned here that, the diffraction spots of SRO and STO are well overlapped, as their lattice parameters are very close. By carefully examining the reciprocal lattices from the STO and BFO, we can derive a large BFO *c*/*a* ratio of ~1.14, corresponding to an out-of-plane lattice parameter *c* ~ 4.45 Å, close to that of tetragonal BFO nanostructure on LaAlO_3_ substrate reported by Zhang et al (*c* = 4.65 Å)^11^. We have checked carefully the lattice space and have found that the *c*-parameter is not very uniform spatially. Most areas show a small *c*-lattice space ~4.5 Å, while there are some locations exhibiting smaller *c* ~ 4.0 to 4.2 Å. The diffraction spots can well reflect the average of the lattice spaces, consistent with our HRTEM image analysis. We also notice that the angle between the *a* and *c* is 89–90°, slightly deviated from the rectangular angle. Therefore, it is safe to conclude that the structure is monoclinic or most likely psuedotetragonal, similar to reported results for BFO/SRO/STO by Chu et al^19^. The large *c*/*a* ratio may be attributed to the big in-plane compressive strain imposed by the substrate, noting the lattice mismatch of 1.7% between strain-free BFO and Nb-STO. Due to the small thickness (~10 nm) of BFO, the strain imposed from the Nb-STO is almost over the whole BFO nanostructures, which may be higher than that with a SRO buffer layer. From the literature, BFO directly deposited on Nb-STO has a much bigger *c*/*a* ratio than that with a SRO layer^19^,^20^, supporting our assumption. Furthermore, our deposition oxygen pressure (2 Pa) is lower than that commonly used for film deposition (10 Pa), which may introduce high density oxygen vacancies and also bring more distortion into the BFO lattice. While the nonuniformity in lattice space may be related to the defects such as dislocations, which can partially relax the local strains, further study is still needed to throw light on the reason for the large lattice distortion in our BFO nanostructures.

**Figure 3:**
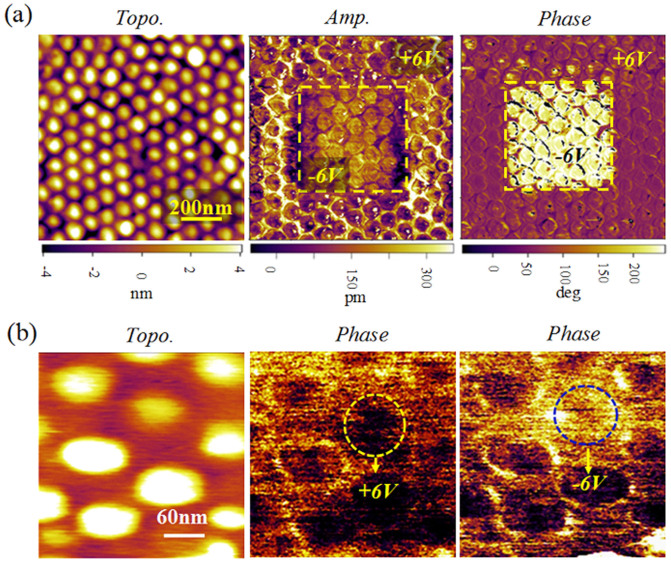
Piezoresponse images for the polarization reversal process in the nanocapacitor arrays. (a) Topological, and piezoresponse amplitude and phase images for the nanocapacitor array, in which in the middle square area was poled downwards (with a bias voltage of −6 V) while the rest part was upwards with a voltage of +6 V; (b) the piezoresponse phase images illustrating the polarization reversal for a selected nanocapacitor dot, which was poled upwards and then downwards using bias of ±6 V, respectively.

To characterize the ferroelectric properties of the nanodots, vertical piezoresponse force microscopy (VPFM) measurements were performed and the results are highlighted in Fig. 3. Fig. 3(a) shows the AFM topography, piezoresponse amplitude- and phase-contrast micrographs for the nanodot arrays. The bright- and dark-contrasts in the phase micrographs correspond to the down-polarization (*P_down_*) and up-polarization (*P_up_*) states, respectively, while the contrast in amplitude piezoresponse is related to the magnitude of the piezoelectric signal. To show the polarization reversal status, the BFO-SRO nanodot array was first electrically poled by applying an external scanning bias at a pre-designed area during the scan, in which the middle area was poled downwards by a reverse voltage of −6 V, and the rest area upwards with a positive voltage of +6 V. From the phase-contrast micrograph, we can observe completely different dark- and bright-contrast area for the dots of different polarization orientations, indicating that the polarizations of the nanodots are reversible under applied electric voltages. From the piezoresponse amplitude-contrast image, it was found that the amplitude for the downward polarization is slightly smaller than that of upward, exhibiting some extent of preferred polarization orientation. In between the two different polarization regions, there are some dots exhibit low piezoelectric amplitude, which may be correspondent to those in domain border region. It is known that for most reports, the domain wall width in BFO thin films is a few nanometers^15^,^19^. However, for our BFO nanodots, the domain structure and configuration can be quite different. The free boundary of nanodots imposes additional mechanical and electric boundary conditions which make the domain structure of nanodots much more complicated than that for thin films. For example, one can observe upward, downward, bubble-like, vortex-like, and stripe-like domain patterns in nanodots. In these cases, the domain wall width, if definable, may be much wider than those in thin films. Therefore, we can only see a bounder region in our nanodot array, instead of a sharp boundary. To demonstrate the reversibility of an individual dot, we applied a pulsed voltage of ±6 V on a selected dot, which produces apparent different contrasts for the two different pulsed voltages, as shown in Fig. 3(b). This confirms that the isolated dot is switchable in polarization by applying a pulsed voltage.

**Figure 4:**
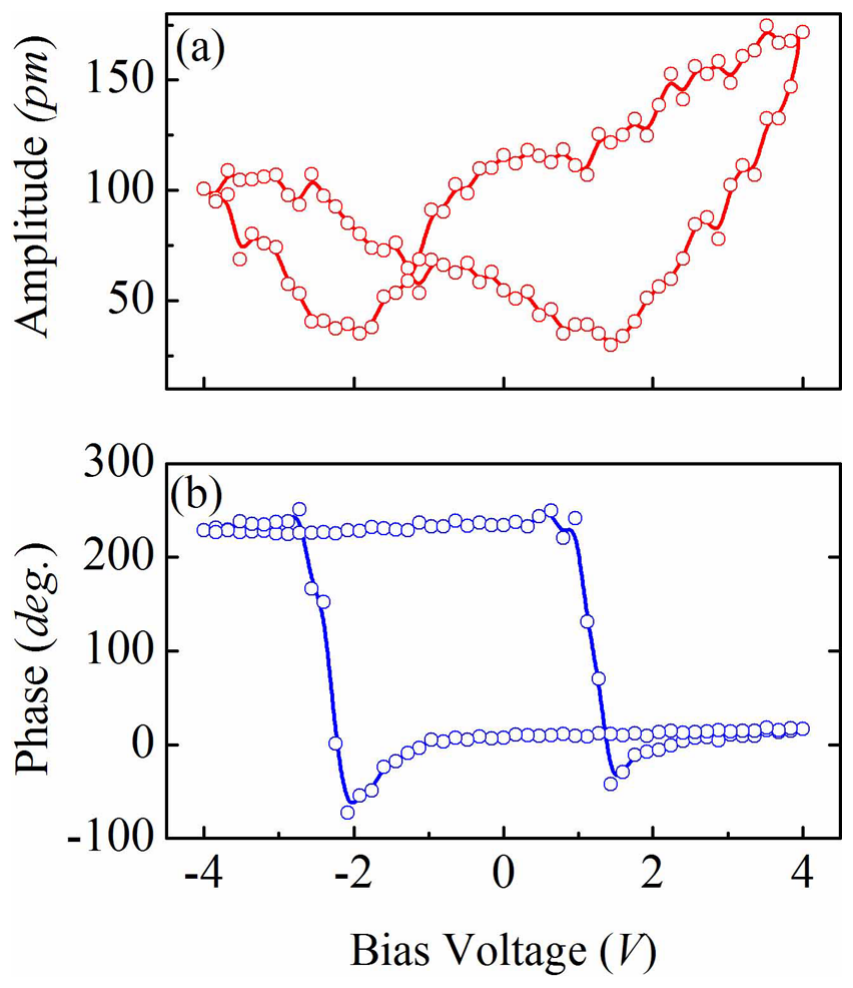
Local piezoresponse hysteresis loops acquired on a single nanocapacitor dot. (a) the amplitude-voltage and (b) phase-voltage piezoresponse hysteresis loops.

To examine the local electric properties of the nanodots, we measured the piezoelectric hysteresis loops on a single nanocapacitor. The piezoresponse phase-voltage hysteresis and the butterfly-like amplitude-voltage loops are displayed in Fig. 4(a) and (b). At a low bias of ~1 V (not shown here), both the amplitude and phase remain rather stable, indicating no reversal process. Once the bias voltage increases beyond 3 V, the switching becomes apparent, producing to a well-developed butterfly amplitude loop and a square phase hysteresis loops at a bias of 4 V. The two asymmetric coercive fields V_+_ = 1.32 V and V_−_ = −2.16 V can be identified, indicating that the polarization reverse is nonsymmetric, as also confirmed by the as-grown states. This may be due to the built-in fields from the work-function difference between the top/bottom electrodes and BFO. From the band structure analysis, we have found that the SRO and NSTO have their work-functions of around 5.2 eV and 4.08 eV, respectively^21^,^22^, which produces an overall theoretical built-in voltage of 1.12 eV. This breaks the equivalence of two polarization states, and provides a strong tendency to alight the domains to a preferred orientation. In additional, oxygen vacancies adjacent the top electrode introduced during the deposition process may contribute to the observed asymmetric polarization states^23^.

**Figure 5:**
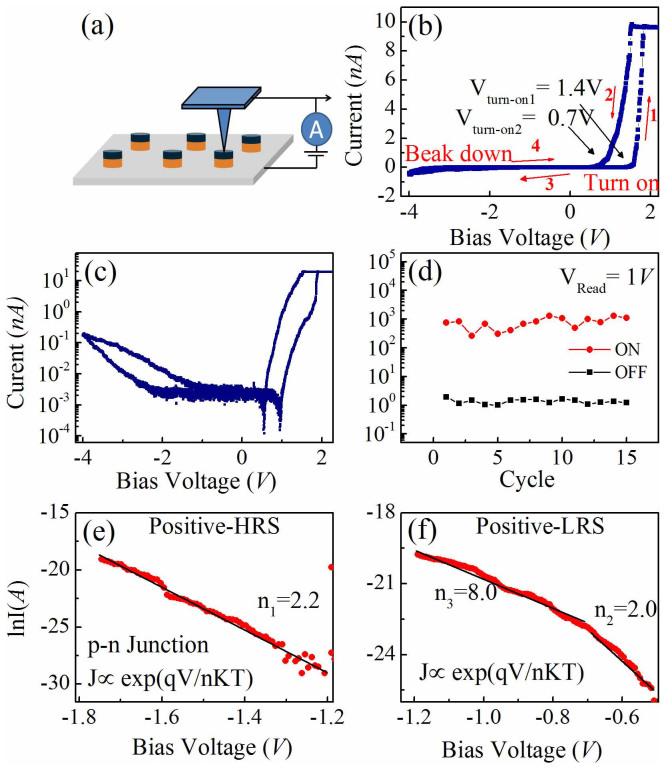
The local conductivity measurement on a single nanocapcitor cell by C-AFM. (a) The schematic diagram for the measurement devices; (b) local I–V curves at a maximum bias voltage of 4 V, showing both a large rectification and a resistive switching behaviors; (c) the replotted *I–V* curve in a semi-logarithmic style; (d) 15-cycles endurance test, with a readout voltage of at 1 V; (e, f) *Ln(I)-V* cures for both HRS (e) and LRS (f) at the positive bias range, which are fitted to the *p-n* conduction exponential relation.

To further examine the resistive property, we look into the local current-voltage (*I–V*) characteristics by CAFM on a single BFO nanodot. The schematic structure of the device is depicted in Fig. 5(a). One observed a hysteresis *I–V* curves in Fig. 5(b) & (c), indicating an apparent resistive switching behavior. To examine the stability of the switching behavior, we performed the I–V sweep at a bias voltage of 4 V for 15 cycles. The ON/OFF current at a reading voltage of 1 V is plotted in Fig. 5(d), which shows more or less stable state with a big R_ON/OFF_ ratio of ~593 up to 15 cycles. Interestingly, a large current rectification behavior can be identified as shown in Fig. 5(b), which can also greatly affect the resistive behaviors. It is well-known that Nb-STO is an *n*-type semiconductor, and BFO with Bi vacancies could be considered as *p*-type semiconductor^21^. Thus, a *p-n* junction can be formed at the BFO/Nb-STO interface, which is most probably the reason for the large current rectification behavior.

To further verify the *p-n* junction behavior, we fitted the semi-logarithmic *I–V* curve to a typical exponential relation for a *p-n* diode, given as^24^:
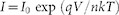
where *I_0_* is the saturated reverse current, *q* is the charge of electron, *k* is the Boltzmann constant, and *T* is the temperature. As shown in Fig. 5(e) and (f), the *ln*(*I*) at both the high resistive state (HRS) and low resistive state (LRS) have nearly linear dependence on V. For an ideal diode, *ln*(*I*) should have a linear relation against V with *n* = 1. However, for a semiconductor diode, at the lower bias, *n* is 2. In our case, the *I–V* curve of high resistive state (HRS) fits the *p-n* relation very well, with *n* ~ 2.2, indicating it is dominated by diode recombination current, as shown in Fig. 5(e). For the low resistive state (LRS), we obtain *n* ~ 2 at low bias range, similar to that of HRS (Fig. 5(f)). However, at higher bias range, *n* is unusually high (*n* ~ 8), indicating the coexistence of semiconductor diode relation at low bias and other mechanism at high bias range for LRS.

**Figure 6:**
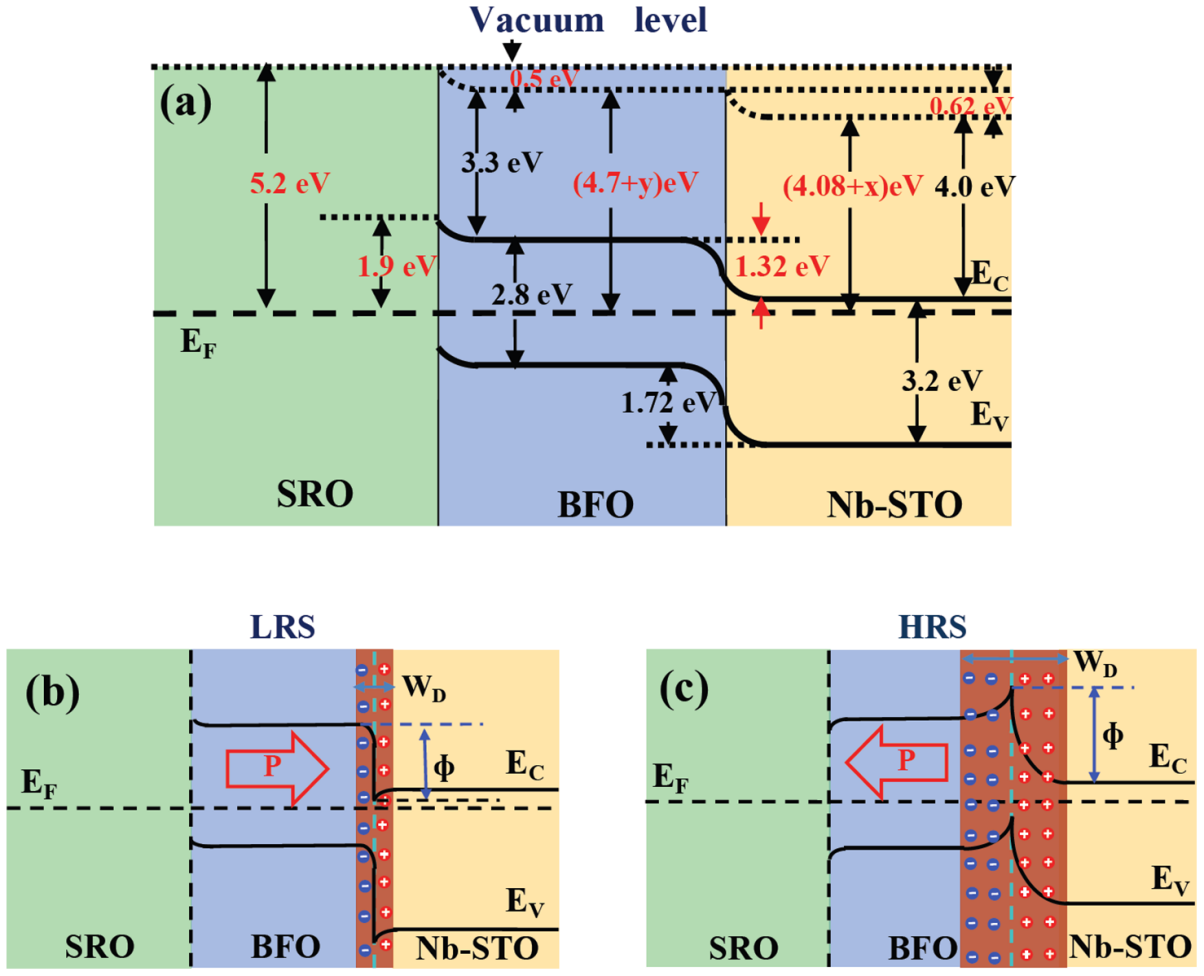
Schematic energy band for the conducting mechanism. (a) Energy band diagram for the whole SRO/BFO/Nb-STO heterojunction; (b, c) present the schematic band diagrams for a *p-n* junction at two different ferroelectric polarization directions, with downward polarization state (b) and upward polarization state (c), corresponding to the LRS and HRS, respectively.

Fig. 6(a) gives a schematic equilibrium band structure of the SRO/BFO/Nb-STO heterojunction, which is a typical staggered energy band diagram. Nb-STO has an energy band gap of 3.2 eV and an electron affinity of 4 eV^25^, while BFO has energy band gap of 2.8 eV and an electron affinity of 3.3 eV^26^. BFO forms a staggered energy band diagram with Nb-STO, while Nb-STO has high conductivity and its Fermi level is close to the bottom of the conduction band. For a non-degenerated semiconductor, the Fermi level is at least 3 *kT* above the energy level of valance band of (E_V_) or 3 *kT* below the energy level of conductive band (E_C_). Therefore the work-function of Nb-STO is deduced to be (4 + 3 *kT + x*) = (4.08 + *x*) eV, where *x* is a small value^21^. BFO has high resistivity, its Fermi level is close to or below the middle of the energy band gap, so the work-function of BFO is deduced to be (3.3 + 1.4 + *y*) eV, where *y* is another small value^21^,^26^. The work-function of SRO is ~5.2 eV^22^. The built-in voltage V_bi_ can be deduced as the difference between the work-functions V_bi(SRO/BFO)_ = 5.2 − (4.7 + *y*) ~ 0.5 V, and V_bi(BFO/Nb-STO)_ = (4.7 + *y*) − (4.08 + *x*) ~ 0.62 V. The two built-in voltages are alighted along the same direction, leading to a big total built-in voltage of ~1.1 V, which can account for the apparent asymmetry and large imprint field of 0.84 V in piezoresponse loops shown in Fig. 4.

From the band structure in Fig. 6(a), the calculated barrier heights by electrons and holes are 1.32 eV and 1.72 eV, respectively. Typically, if the barrier height for holes is 0.2 eV higher than that for electrons, the hole current will be approximately a factor of 10^4^ smaller than the electron current^27^. Therefore, the conductive behaviors are mainly dominated by the major charge carrier (electrons). The barrier height for the electrons is 1.32 eV, corresponding to a turn-on voltage of 1.32 V for an ideal *p-n* junction. From the I–V curve in Fig. 5(b), we can evaluate the two different turn-on voltages of 1.4 V for the HRS and 0.7 V for the low LRS, respectively. This is more or less deviated from the ideal turn-on voltage of 1.32 V, likely due to the band modulation by ferroelectric polarizations.

The observed resistive switching behavior in the SRO/BFO/Nb-STO heterostructures can be accounted for by the ferroelectric polarization modulation on both the width of depletion region and the height of potential barrier at the BFO/Nb-STO interface, as illustrated by our schematic *p-n* junction model^28^,^29^. If no external polarization influences, the depletion width across the BFO/Nb-STO may stay at a certain degree after reaching the dynamic equilibrium state. At downward polarization, the negative majority electron carriers in the *n*-type Nb-STO are attracted by the positive bound charges and migrate away from the interface, resulting in a decrease in the depletion width^24^,^30^, as schematically shown in Fig. 6(b). In contrast, when the polarization is aligned upwards, the negative majority electron carriers in the *n*-type Nb-STO are repelled by the negative bound charges at the BFO/Nb-STO interface, which increases the depletion width, as shown in Fig. 6(c). The depletion region also induces an energy band bending leading to the change of potential barrier height at the interface^31^, resulting in the variation in turn-on voltages at the two different polarization orientations as exhibited in Fig. 6(b). This agrees well with the experimental values of the turn-on voltage of 0.7 V for LRS and 1.4 V for HRS, respectively (shown Fig. 5(b)). Therefore, the resistive switching behavior in SRO/BFO/Nb-STO heterostructure could be attributed to the modulation of both the depletion width and the potential barrier height by polarization reverse inside the BFO film. It is also worthy of mention that the SRO/BFO interface can play important role in the current rectifying behavior^23^. However, for our SRO/BFO nanostructures and heterostructures deposited using the similar parameters, we are not able to obtain such big current rectification ratio (~1000) while it is rather commonly observed for BFO/Nb-STO heterostructures^21^,^24^. Furthermore, if the p-n junction behavior is from the BFO/SRO interface, it would produce a backward p-n junction *I–V* behavior instead of the observed forward p-n junction behavior. Therefore, the observed large current rectification is more likely dominated by the BFO/Nb-STO interface.

In summary, well-ordered SRO/BFO/Nb-STO nanocapacitor arrays have been successfully fabricated by PLD in combination with the ultrathin AAO stencil masking method. The BFO nanodots show well-epitaxial tetragonal-like structure with large *c*/*a* ratio due to the substrate induced compressive strain. The nanocapacitor arrays show well reversible ferroelectric polarization. Moreover, these BFO nanodots present an apparent resistive switching behavior along with a diode-like rectifying current-voltage characteristic, which are accounted for by a polarization modulated p-n junction model. These results indicate that the nanocapacitor arrays have potential for nano-device applications.

## Methods

### Fabrication of nanocapcitor array

The fabrication procedure of BFO/SRO nanostructures is illustrated in Fig. 1(a). First, the BFO nanodots were epitaxially grown on a conductive 0.7 wt% Nb-doped (100) SrTiO_3_ (Nb-STO) substrate through an AAO template (Fig. 1(b)) by PLD using a KrF excimer laser (λ = 248 nm) and an ambient temperature of 500°C and a low oxygen pressure of *2 Pa*. Subsequently, the SRO top electrodes were deposited through the AAO template on top of BFO at the same temperature. Finally, the AAO mask was lifted-off by mechanical method, leaving the well-ordered nanocapacitor arrays as shown in Fig. 1(c). Here, the AAO templates with ~60 nm pore size were fabricated by a two-step anodization of electropolished Al sheets.

During the procedure, the first anodization of Al sheet in 0.3 M H_2_C_2_O_4_ solution was conducted for 24 h at 5°C, then the anodized Al sheet was completely removed in an aqueous acid mixture of H_3_PO_4_ and CrO_3_ (6.0 wt% and 1.8 wt%) at 45°C for 12 hrs. The second anodization was carried out for 5 mins at 5°C to get well ordered pores, followed by an etching process in CuCl_2_ at 10°C which detached the alumina layer from the Al sheet. In sequence, the barrier layer was removed during the pore widening process with 5 wt% H_3_PO_4_ at 35°C for 30 min. Finally, we obtained ~300 nm-thick AAO membranes that can be transferred to various substrates (e.g. Nb-STO).

### Structure and nanoscale electric characterizations

The crystallineities of Nb-STO/BFO/SRO nanocapacitors were characterized by X-ray diffraction (PANalytical X′ Pert PRO). The cross-section images were illustrated by high resolution transmission electron microscopy (HRTEM, JOEL-2011). The topology was examined by atomic force microscopy (AFM). The ferroelectric domain structures were probed by piezoresponse force microscopy (PFM) (Cypher, Asylum Research) using a dual-frequency resonant-tracking technique (DART), and the local I-V curve and resistive switching loops were obtained by fixing conductive atomic microscopy (CAFM) with Pt/Ti coated conductive AFM probes (Nanosensor), where the AFM probe were fixed at a certain point, and then sweeping the dc bias upwards and downwards between bias voltage of *±V_m_* for certain cycles.

## Author Contributions

L.N.Z. conducted the data acquisition and helped draft the manuscript, Z.X.L. and G.T. participated in the sample fabrication and X.R.D. measurement. F.Y.Z., X.S. and W.Z.L. carried out the PFM and CAFM measurement. K.R.H. and Z.Z. contributed to the AAO preparation. S.J.W., X.B.L., M.H.Q. and M.Z. contributed to the data interpretation. J.Y.D. contributed to the TEM observation. X.S.G. & J.M.L. contributed to the data interpretation and manuscript writing. X.S.G. supervised the research.
